# Sociocultural attitudes towards appearance questionnaire-4-revised (SATAQ-4R): validation in a community sample of Norwegian adolescents

**DOI:** 10.1186/s40337-024-01151-4

**Published:** 2024-11-28

**Authors:** Selma Øverland Lie, Rafael Valdece Sousa Bastos, Christine Sundgot-Borgen, Line Wisting, Camilla Lindvall Dahlgren

**Affiliations:** 1 Regional Department of Eating Disorders, Division of Mental Health and Addiction, Oslo University Hospital-Ullevål, Oslo, Norway; 2 São Francisco University, Paulista, Brazil; 3 Department of Psychology, University of Oslo, Oslo, Norway; 4 Department of Psychology, Oslo New University College, Oslo, Norway

**Keywords:** Validation, Internalization, Sociocultural pressures, Disordered eating

## Abstract

**Background:**

Negative body image and disordered eating are common among adolescents and young adults. The Sociocultural Attitudes Towards Appearance Questionnaire-4-Revised (SATAQ-4R) captures the internalization of societal appearance ideals and perceived pressures from others but has not been validated in a Norwegian adolescent population.

**Methods:**

The current study explored the factor structure of SATAQ-4R in a sample of adolescent Norwegian males and females (*n* = 1558, mean age 17.04 ± 0.95) using confirmatory factor analysis (CFA) for a 6- and 7-factor structure in females, and a 7-factor structure in males. Correlations between subscales, internal consistency and reliability, and comparisons with convergent measures (disordered eating, body mass index, negative influence of social media) were explored.

**Results:**

The CFA supported a 7-factor structure of the SATAQ-4R for both males and females. Internal consistency and reliability were acceptable. SATAQ-4R subscales largely correlated with disordered eating and additional convergent measures.

**Conclusions:**

Results confirm the structure and reliability of the SATAQ-4R in a Norwegian adolescent population. The subscales showed good convergent validity, and high scores on internalization and societal pressures were related to higher levels of disordered eating and negative social media influence. The Norwegian version of the SATAQ-4R thus demonstrates good psychometric properties in adolescent males and females, and is well suited to capture internalization and sociocultural pressures that particularly affect adolescents. Results highlight the need to continue working towards reducing adverse internalization and improving body image among adolescents.

## Abstract (plain language summary)

Appearance pressures and internalization of appearance ideals are empirically supported risk factors for body dissatisfaction and disordered eating. Having validated tools to measure these risk factors is essential, particularly for young people, as adolescence itself presents an increased risk for the development of eating disorders. The SATAQ-4R is a self-report questionnaire designed to measure the internalization of western beauty standards, and pressure to conform to these. Although the measure has been used in Norwegian research, it hasn’t been validated yet. This study aimed to test how well the SATAQ-4R works with Norwegian teenagers. The results showed that it effectively captures the internalization of societal expectations and pressures that particularly affect adolescents.

## Background

Body image is a multidimensional construct encompassing the thoughts, feelings, and behaviors of an individual related to their own appearance, and represents aspects related to both body satisfaction and concern [1–4]. The prevailing societal expectations regarding body appearance, such as all bodies looking lean and strong, pose a threat to the development of healthy body images in adolescents [5–8]. Among several components of body image, body dissatisfaction refers to a negative evaluation of one’s body [9], and is a well-known risk and maintenance factor for eating disorders (EDs) [10]. Eating disorders are psychiatric disorders characterized by dysregulated food intake, and associated behaviors and cognitions relating to body image, weight, and shape [11]. Such cognitions can for example be that body weight has an undue influence on self-worth or that having a slim figure is more important than feeling strong. While clinical EDs require meeting strict diagnostic criteria, disordered eating (DE), a common phenomenon among adolescents [12], encompasses various forms of maladaptive eating behaviors such as frequent dieting, loss of control of eating, and various compensatory behaviors to prevent weight gain that can negatively impact health [13, 14]. Moreover, DE behaviors such as dieting and binge eating are common risk factors for clinical ED onset) [15, 16].

Adolescents are particularly vulnerable to developing issues related to food, weight, and body shape [17]. Globally, the prevalence of DE in children and adolescents is approximately 30% in girls and 17% in boys, with higher rates observed in older adolescents compared to younger age groups [14, 18]. Among Norwegian adolescents, the prevalence of DE ranges from 13 to 71%, with 4–32% reporting body dissatisfaction [19, 20]. A recent study found that 5% of male and 33% of female Norwegian adolescents were classified as struggling with disordered eating behaviors [21].

Both EDs and DE can significantly impair physical health and disrupt psychosocial functioning, often leading to disability and high individual and societal costs [22]. Given the high prevalence of these issues and associated negative outcomes, considerable attention has been directed towards understanding the mechanisms contributing to the development of DE and EDs [23]. One prominent framework is the tripartite influence model, which explains how social influences from parents, peers, media (including social media) and significant others (e.g., athletic coaches, teachers) serve as critical avenues for the reinforcement of appearance ideals. These social pressures may lead to the internalization of body ideals, and the subsequent pressure to conform to these standards [24–26]. Body ideal internalization is proposed as a key psychological mechanism for understanding the development of body dissatisfaction [27]. Pressures to conform to appearance standards, particularly those aligned with Western beauty ideals, lead to the internalization of these body ideals. This internalization fosters dissatisfaction with one’s own body, which can ultimately result in disordered eating behaviors.

Gender differences in body image and appearance ideals have previously been reported, with lower levels of body appreciation in females compared to males [28], though some research indicates body dissatisfaction across genders [29]. In terms of body appearance ideals, girls seem to a higher degree to internalize a thin ideal than boys, who tend to internalize a lean and muscular ideal [30]. Moreover, in support of previous research, a recent study on Norwegian adolescents revealed that internalization of the *muscular body ideal* was weakly associated with ED pathology in both boys and girls. In contrast, internalization of the *thin ideal* showed a weak association with ED pathology in boys, but a strong association in girls. This is not surprising, as girls are disproportionately subjected to thin ideal standards. Indeed, this study also reported that boys scored significantly higher than girls on the muscular subscale. Taken together, this highlights the need for gender-specific measurements of both internalization and sociocultural appearance pressure [31].

To assess the role of appearance internalization and pressure to conform to cultural standards, the Sociocultural Attitudes Towards Appearance Questionnaire (SATAQ) was developed. Since the publication of the original version [32], numerous revisions have been undertaken to create versions that more accurately reflect the evolving cultural perceptions of body ideals in males and females [33–35]. The most recent version of the SATAQ, the SATAQ-4R [35], has been adapted to better capture the desire for muscularity for aesthetic reasons. It focuses on cognitive rather than behavioral aspects of internalization, includes an assessment of general appearance internalization in addition to internalization of thin and muscular ideals in specific, and incorporates a pressure subscale related to significant others. Additionally, this current revision offers separate versions for males (28 items) and females (31 items). Both versions of the SATAQ-4R have demonstrated strong internal consistency, with Cronbach’s alpha values of ≥ 0.82 and ≥ 0.75 for each sub-scale, respectively, based on college student samples [35]. The subscales of the SATAQ-4R have been negatively associated with self-esteem and positively associated with ED symptoms and body dissatisfaction in both males and females [35]. These findings have been replicated among Italian men and women [36] and Turkish women [37]. The original 7-factor structure identified in American college students (Schaefer et al., 2017) has been confirmed within the Italian sample [36]. When validating the instrument on Turkish women, a 6-factor structure was observed. This could indicate that cultural differences affect the factor structure, and should be examined when validated in new groups [37].

The SATAQ-4R has primarily been validated in adult samples, with limited validation among adolescents. Schaefer et al. [35] included a sample of young adolescent females (10–14 years), and a recent study by Huang, Liu [38] validated the measure in male and female 13–18-year-olds. Notably, these studies revealed diverging factor structures. The significant differences in physical, social, and mental constructs between adults and adolescents [39] necessitate age-specific validation to ensure the measure’s reliability and validity in younger age groups. Sources of pressure may vary across different stages of adolescence. While family tends to exert the most influence on younger adolescents, older adolescents and young adults are more likely to experience pressures from a broader range of significant figures, including teachers, employers, and coaches. Moreover, peer pressure experiences may shift with age and pubertal development. Social media also becomes increasingly impactful during mid to late adolescence, as most platforms are restricted to users above a certain age threshold In Norway, the SATAQ-4R has been applied in research including younger [40] and older [31, 41, 42] adolescents with observed Cronbach’s alpha values ranging from ≥ 0.76 in boys to ≥ 0.90 in girls. The SATAQ-4R has also been used in two young adult Norwegian samples [43–45] with Cronbach’s alpha values from ≥ 0.79 and ≥ 0.81 in males and females. However, existing studies have not focused explicitly on how males and females score across the different subscales [31, 40, 41, 46], and the instrument has never been validated in a Norwegian adolescent sample. Therefore, an examination of the instrument’s suitability for this population is warranted.

This study aimed to seek evidence of validity of the measure in a sample of Norwegian adolescents, incorporating gender-specific analyses. Given that previous validation studies have identified both 6- and 7-factor models for the female version of the measure, we explored both models to determine the best fit for our data using confirmatory factor analysis. For the male version, fit statistics for the 7-factor model were examined in accordance with previous findings. Based on existing literature, we anticipated moderate levels of internalization and appearance-related pressures in both males and females, which we expected to be associated with disordered eating. In line with existing literature, we also anticipated gender differences in internalization types, such as muscular internalization being more prevalent among boys, and internalization of the thin ideal being more prevalent in girls. Furthermore, we anticipated good internal consistency and reliability of the various subscales, and expected to find correlations between the different types of internalization and pressures among both male and female adolescents as these have been correlated in previous SATAQ-4 validations.

## Methods

### Procedure and sample

The data for this study were obtained as part of a broader investigation into the prevalence and correlates of EDs among Norwegian adolescents, predominantly aged 16–19 years (range = 16–23) [21]. Participant recruitment was concentrated in large urban areas of the country. Data collection took place from November 2020 to May 2021, using an online survey distributed through school channels. Participation was voluntary, with informed consent obtained electronically from students 16 years or older. As a main rule in Norway, minors above the age of 16 can consent to participate in research without parental approval. The survey explored dietary habits, body and weight concerns, loneliness, appearance attitudes and pressure, quality of life, social media use, and non-suicidal self-injury (see 21, 47). Preceding the survey, students, parents, and teachers received detailed information about the study through multimedia and digital outreach. Ethical clearance was secured from the Norwegian Committee for Medical and Health Research Ethics (Ref ID 116178) and the local Data Protection Authority. The study is registered in the Open Science Framework (Identifier: DOI 10.17605/OSF.IO/5RB6P).

### Assessment

#### Sociodemographic information

The online survey solicited self-reported data on weight and height to determine Body Mass Index (BMI in kg/m²), along with participants’ age, gender, ethnic background, educational institution, academic level, and study program.

#### The sociocultural attitudes towards appearance questionnaire-4 revised (SATAQ-4R)

The SATAQ-4R [35] evaluates individuals’ internalization of societal standards of appearance and attractiveness and the perceived pressures to conform to these standards from various social sources, including family, peers, partners, and media. The instrument provides gender-specific iterations, comprising 31 items for females and 28 for males. It employs a 5-point Likert response scale ranging from “Strongly Disagree” to “Strongly Agree” and is organized into seven subscales: (1) *Internalization: Thin/low body fat*; (2) *Internalization: Muscular*; (3) *Internalization: General attractiveness*; (4) *Pressures: Family*; (5) *Pressures: Media*; (6) *Pressures: Peers*; and (7) *Pressures: Significant others*. Each subscale’s mean score is computed, with higher scores indicating greater levels of internalization and perceived pressures. The Norwegian adaptation of the SATAQ-4R has been validated, demonstrating robust psychometric qualities [43].

#### The eating disorder examination questionnaire short (EDE-QS)

The EDE-QS [48] is a short version of the Eating Disorder Examination Questionnaire (EDE-Q; 49) designed to evaluate ED related thoughts and behaviors from the previous week. The EDE-QS features 12 items rated on a 4-point Likert scale (0 = 0 days, 1 = 1–2 days, 2 = 3–5 days, 3 = 6–7 days). Aggregate scores range from 0 to 36, with higher totals suggesting increased symptom severity. The measure has demonstrated good internal consistency, test-retest reliability, and convergent validity in individuals with probable and non-probable EDs [48, 50]. Its brevity and psychometric properties underpin the instrument’s potential as an effective screening instrument for EDs in in non-clinical samples [48]. The optimal balance of sensitivity and specificity is achieved with a cut-off score of 15 [50], although adjustments to lower scores are permissible to capture those at significant risk of developing EDs. In this study, a cut-off score of 13 was used to indicate problematic eating or eating concerns (see 21). In addition to the total score, item scores for item 11: “*Has your weight or shape influenced how you think about (judge) yourself as a person?*“ and item 12: “*How dissatisfied have you been with your weight or shape?”* were used as measures of convergent validity as they target concepts expected to be related to the SATAQ-4R subscales. The translation of the English EDE-QS to Norwegian was carried out in line with the Norwegian version of the EDE-Q [51]. Cronbach’s alpha obtained in the current sample was .90, indicating excellent internal consistency.

#### The eating disorder assessment for DSM-5 (EDA-5)

The EDA-5 interview [52] was used to assess EDs following DSM-5 criteria. Operationalization of the diagnostic criteria as applied in the EDA-5 is presented in detail in Walsh et al. [53] and in Dahlgren et al. [21]. Recent findings from the Norwegian validation study confirm that the Norwegian EDA-5 can produce ED diagnoses both effectively and accurately [54]. In this study, the EDA-5 was used for a subsample of 99 participants (87 girls and 12 boys) who scored at or above the EDE-QS cut-off.

#### Social media influence

Participants were prompted to indicate (Yes/No) whether their use of social media contributed negatively to their appearance perception, via the question «*Do any of the social media platforms you use have a negative influence on how you think about your appearance?*» The selection of social media platforms reflected the prevalent usage in Norway in 2020, as identified by iPSOS market research, encompassing Instagram, Snapchat, TikTok, YouTube, Pinterest, X/Twitter, Facebook, Reddit, Twitch, Jodel, and Tinder.

### Statistical analysis

To seek structural evidence for the seven-factor structure of the SATAQ-4R, two confirmatory factor analyses were conducted for males and females separately, using *lavaan* R package [55] with a Diagonally Weighted Least Squares (DWLS) estimator. The DWLS estimator was employed due to its superiority in generating precise estimates for inter-factor correlations and factor loadings compared to Maximum Likelihood estimation. Additionally, DWLS is preferred for its enhanced control over Type I error rates [56]. The goodness of fit of the model was assessed using the following fit statistics: chi-squared (χ²), Tucker-Lewis Index (TLI); comparative fit index (CFI); root mean square error of approximation (RMSEA); the standardized root mean square residual (SRMR). In line with recommendations [57], the cut-off values close to 0.95 for TLI and CFI, RMSEA < 0.06, and SRMR < 0.08 were used to indicate a good fit for any given model. We also compared the 6-factor structure found in the literature [37] with the 7-factor structure in women using the Tetrad Fit Index (TFI; 58). The difference between the 6-factors model is the concatenation of *“Pressures: Peers*” items with *“Pressures: Significant others”* items. The TFI is based on the vanishing tetrads [59], and was used because the CFA model views a causal relationship between items and constructs, where the level of the latent variable is the cause of the variation in the level of the indicator [60]. The TFI is a model comparison fit that considers this causal relationship. TFI values vary from 0 (fits as good as possible) to 1 (fits as bad as possible). The model that presents the lowest fit will be considered the best model. The TFI was calculated using the bifactor R package [61].

A receiver operating characteristic (ROC) analysis was used to evaluate the True and False Positive Percentage (sensitivity and specificity, respectively) of the SATAQ-4R to identify clinical ED diagnosis (diagnosis yes/no based on the EDA-5 interview). True Positive Percentage represents the proportion of positive instances that are correctly classified by the model, while the False Positive Percentage represents the proportion of positive instances that are classified incorrectly by the model [62]. We used Youden’s J statistic [63] to determine the best threshold (i.e., predictive probabilities) cut-off, where the optimal cut-off is the threshold that maximizes the distance to the identity line (the diagonal line in the figure). In this analysis, we consider a small subsample of female adolescents (*N* = 87) of the larger sample (*N* = 1558) who scored above the EDE-QS cut-off, and agreed to participate in the EDA-5 interview. EDA-5 interview data was only available for 12 male adolescents, so we did not perform a ROC analysis on males due to low n. 49% of those who were interviewed received an ED diagnosis (see 21). A two-tailed p-value was calculated using the z-distribution, in addition to the confidence interval of the Area Under the Curve (AUC). Streiner and Cairney [64] show that the accuracy of tests with an AUC between 0.50 and 0.70 is low; an accuracy between 0.70 and 0.90 is moderate, while an AUC over 0.90 indicates high accuracy. Thus, only subscale factors with an AUC above or equal to 0.70 were considered for further classification analysis. Only participants who responded to all questionnaire items were considered, and responses from male and female participants were separated in this analysis.

Internal consistency for each of the SATAQ-4R subscales for males and females was evaluated using Cronbach’s alpha (α), Revelle’s omega, and greatest lower bound (see 65). After testing normality using Shapiro Wilk’s test, Spearman’s rank (rho, ρ) correlations were used to assess correlations between subscales and to investigate correlations with convergent validity measures EDE-Qs total score and selected item scores, and BMI. These were interpreted as very weak (ρ = 0.00-0.19), weak (ρ = 0.20–0.39), moderate (ρ = 0.40–0.59, strong (ρ = 0.60–0.79), very strong (ρ = 0.80-1.0). Point-Biserial correlations were used to estimate the association between SATAQ-4R subscale scores and the dichotomous variable negative influence of social media. Due to the number of comparisons, we used a significance level of *p* < .01. All statistics were calculated using R version 4.3.2 [66].

## Results

### Participant characteristics

1558 individuals participated in the study, 53% were female and 47% were male. Age ranged from 16 to 23 years (M 17.04 SD 0.95) with 99.2% of the participants falling within the age range 16–19. In total, 14.2% were registered as having an immigrant background (13.9% of the girls and 14.5% of the boys). 91% of both male and female participants reported being born in Norway. The average BMI for the total sample ranged from 14 to 62, with a total mean of 21.8 (SD = 3.4); 21.7 (SD = 3.5) for females, and 21.8 (SD = 3.3) for males. Of females, 33.2% of the participants scored above the EDE-QS cut-off score of 13 indicating problematic eating or eating concerns. Among males, only 4.79% scored above cut-off. The estimated prevalence of DSM-5 EDs in the total sample was 9.4%, with other specified feeding or eating disorder (OSFED) diagnoses being the most common (5.6%). In girls, the estimated prevalence of EDs was 16.4%. The underrepresentation of male participants in the diagnostic interviews did not allow for an independent analysis of their diagnostic data.

### Factor analyses

The 7-factor model in the female sample revealed a good fit: χ² (413, *N* = 796) = 877.67, *p* < .001; CFI = 0.988; TLI = 0.986; RMSEA = 0.038 (CI 90% 0.034–0.041); SRMR = 0.05. The factor loadings are reported in Table 1.

**Table 1:** Factor loadings of the 7-factor SATAQ-4R model in the female adolescent sample

SATAQ-4R Scale	Internalization			Pressure			
Item	Thin/Low body fat	Muscular	General Attractiveness	Family	Peers	Significant Others	Media
Item 3	0.741						
Item 6	0.890						
Item 11	0.744						
Item 13	0.841						
Item 1		0.868					
Item 4		0.957					
Item 8		0.854					
Item 10		− 0.302					
Item 15		0.734					
Item 2			0.523				
Item 5			0.807				
Item 7			0.516				
Item 9			− 0.725				
Item 12			0.690				
Item 14			− 0.496				
Item 16				0.860			
Item 17				0.809			
Item 18				0.819			
Item 19				0.594			
Item 20					0.680		
Item 21					0.910		
Item 22					0.821		
Item 23					0.893		
Item 24						0.786	
Item 25						0.840	
Item 26						0.846	
Item 27						0.894	
Item 28							0.792
Item 29							0.876
Item 30							0.797
Item 31							0.893

Note All factor loadings yield *p* < .001. Item 9, 10, and 14 are reverse scored and therefore yield negative factor loadings

The confirmatory factor analysis for the male sample also showed good fit statistics for the instrument: χ² (329, *N* = 703) = 834.328, *p* < .001; CFI = 0.982; TLI = 0.979; RMSEA = 0.047 (CI 90% 0.043–0.051); SRMR = 0.063. The factor loadings are shown in Table 2.

**Table 2:** Factor loadings of the SATAQ-4R 7-factor model in the male adolescent sample

SATAQ-4R Scale	Internalization			Pressure			
Item	Thin/Low body fat	Muscular	General Attractiveness	Family	Peers	Significant Others	Media
Item 2	0.672						
Item 4	0.994						
Item 1		0.833					
Item 3		0.892					
Item 5		0.754					
Item 8		0.718					
Item 6			0.912				
Item 7			0.859				
Item 9				0.521			
Item 10				0.661			
Item 11				0.342			
Item 12				0.744			
Item 13				0.565			
Item 14					0.866		
Item 15					0.882		
Item 16					0.913		
Item 17					0.598		
Item 18						0.843	
Item 19						0.879	
Item 20						0.641	
Item 21						0.898	
Item 22						0.898	
Item 23							0.876
Item 24							0.662
Item 25							0.875
Item 26							0.712
Item 27							0.925
Item 28							0.937

Note All factor loadings yield *p* < .001

Running a model comparison between 6 and 7 factors in the female sample using the TFI, there was evidence in support of the 7-factor structure in female adolescents (TFI = 0. 1363424), compared to the 6-factor structure (TFI = 0.137163).

**Table 3:** Mean scores of the sociocultural attitudes towards appearance questionnaire-4-revised (SATAQ-4R) for females and males

SATAQ-4R Subscale	Female, M (SD)	Male, M (SD)
Internalization: Thin/low body fat	3.30 (1.08)	1.84 (0.95)
Internalization: Muscular	2.55 (0.91)	3.49 (1.00)
Internalization: General attractiveness	4.25 (0.62)	3.34 (1.07)
Pressures: Family	2.19 (1.05)	1.86 (0.71)
Pressures: Peers	2.40 (1.10)	2.44 (1.06)
Pressures: Significant others	1.85 (1.01)	1.74 (0.9)
Pressures: Media	3.43 (1.15)	2.12 (1.10)

### SATAQ-4R mean scores

For SATAQ-4R Female, the mean scores for the different subscales ranged from 1.85 (*Pressures: Significant others*) to 4.25 (*Internalization: General attractiveness*). The scores on SATAQ-4R Male were generally lower and ranged from 1.74 (*Pressures: Significant others*) to 3.49 (*Internalization: Muscular*). The *Internalization: Muscular* scores were higher for males than females. Statistical mean score comparisons are reported elsewhere [31]. See Table 3 for an overview of all scores for males and females.

### ROC analyses

The ROC analysis of the female adolescents using the seven-factor model revealed that all factors had low accuracy (AUC between 0.50 and 0.70) in distinguishing between those with and without EDs (*Internalization: Thin/Low body fat* AUC = 0.678; *Internalization: Muscular* AUC = 0.535; *Internalization: General attractiveness* AUC = 0.526; *Pressures: Family* AUC = 0.514; *Pressures: Peers* AUC = 0.559; *Pressures: Significant others* AUC = 0.517; *Pressures: Media* AUC = 0.537).

### Internal consistency and intercorrelations between subscales

Internal consistency and reliability for the seven subscales of SATAQ-4R female version was acceptable according to Cronbach’s alpha, total omega, and GBL indexes (0.87–0.97). For the seven subscales of the male SATAQ-4R version, all three fit indices also showed acceptable consistency and reliability of the scales (0.81–0.97). See Table 4 for an overview of all indices.

**Table 4:** Reliability of the sociocultural attitudes towards appearance questionnaire-4-revised (SATAQ-4R) subscales for male and female adolescents

Subscale	Cronbach’s Alpha, α^a^		Revelle’s Omega Total^a^		Greatest Lower Bound	
	Female	Male	Female	Male	Female	Male
Internalization: Thin/low body fat	0.91	0.86	0.92	-	0.88	-
Internalization: Muscular	0.90	0.91	0.92	0.92	0.90	0.89
Internalization: General attractiveness	0.88	0.91	0.91	-	0.87	-
Pressures: Family	0.91	0.82	0.94	0.91	0.87	0.83
Pressures: Peers	0.91	0.91	0.93	0.92	0.88	0.90
Pressures: Significant others	0.95	0.95	0.96	0.97	0.91	0.94
Pressures: Media	0.93	0.96	0.95	0.98	0.91	0.97

Note Omega total and Greatest Lower Bound cannot be calculated with only two items in scale

^a^ Alpha and Omega calculated with a polychoric correlation matrix, Greatest Lower Bound based on Pearson correlations

**Fig. 1:**
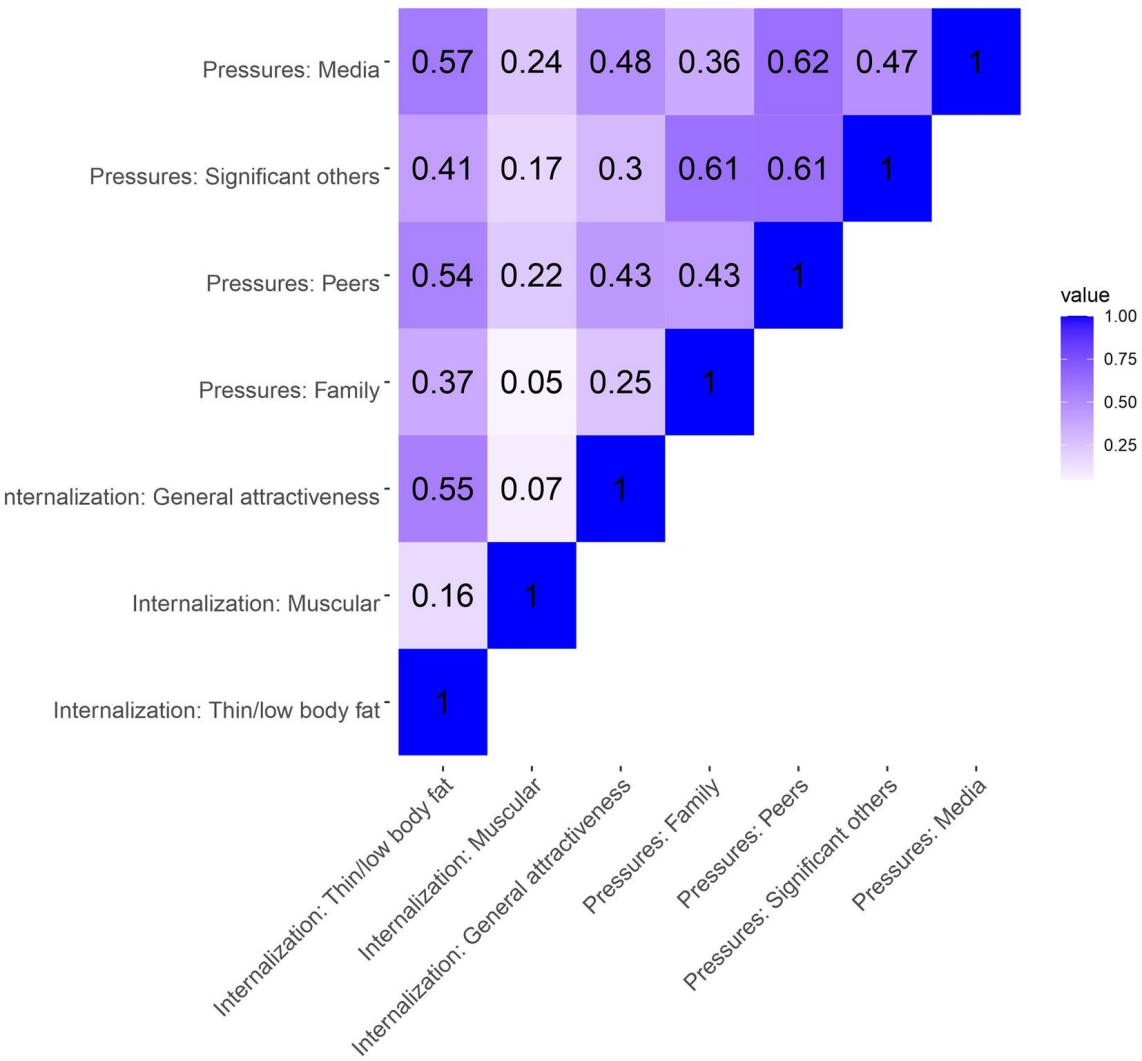
Intercorrelations (Spearman’s rho) between SATAQ-4R subscales for female adolescents. Note. Darker color indicates stronger correlations (closer to 1). SATAQ-4R = sociocultural attitudes towards appearance questionnaire 4 revised

**Fig. 2:**
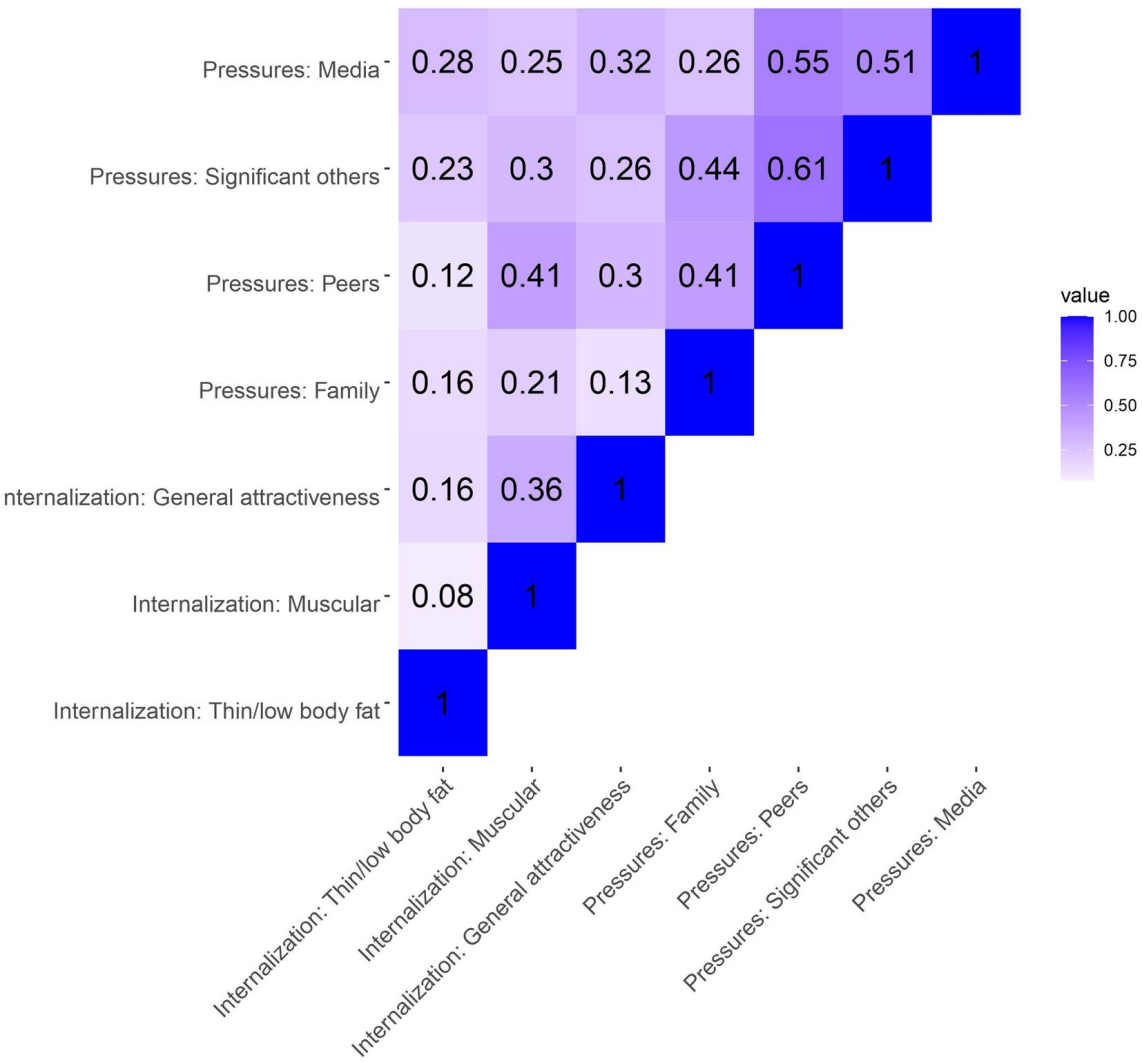
*Intercorrelations (Spearman’s rho) between SATAQ-4R subscales for male adolescents. note.* darker color indicates stronger correlations (closer to 1). SATAQ-4R = sociocultural attitudes towards appearance questionnaire 4 revised

Except for *Internalization: Muscular* with *Internalization: General attractiveness* and *Pressures: Family*, all subscale intercorrelations were significant at the *p* < .01 level. Significant correlations ranged from very weak (*Internalization: Muscular* and *Internalization: Thin/low body fat*) to strong (*Pressures: Media* and *Pressures: Peers*), with Spearman’s rho’s between 0.16 and 0.62) for females. For males, there were significant positive correlations between all subscale, ranging from very weak (*Internalization: Muscular* and *Internalization: Thin/low body fat*) to strong (*Pressures: Significant others* and *Pressures: Peers*), with Spearman’s rho’s between 0.08 and 0.61. See Figs. 1 and 2 for the correlation matrices.

### Convergent validity

**Fig. 3:**
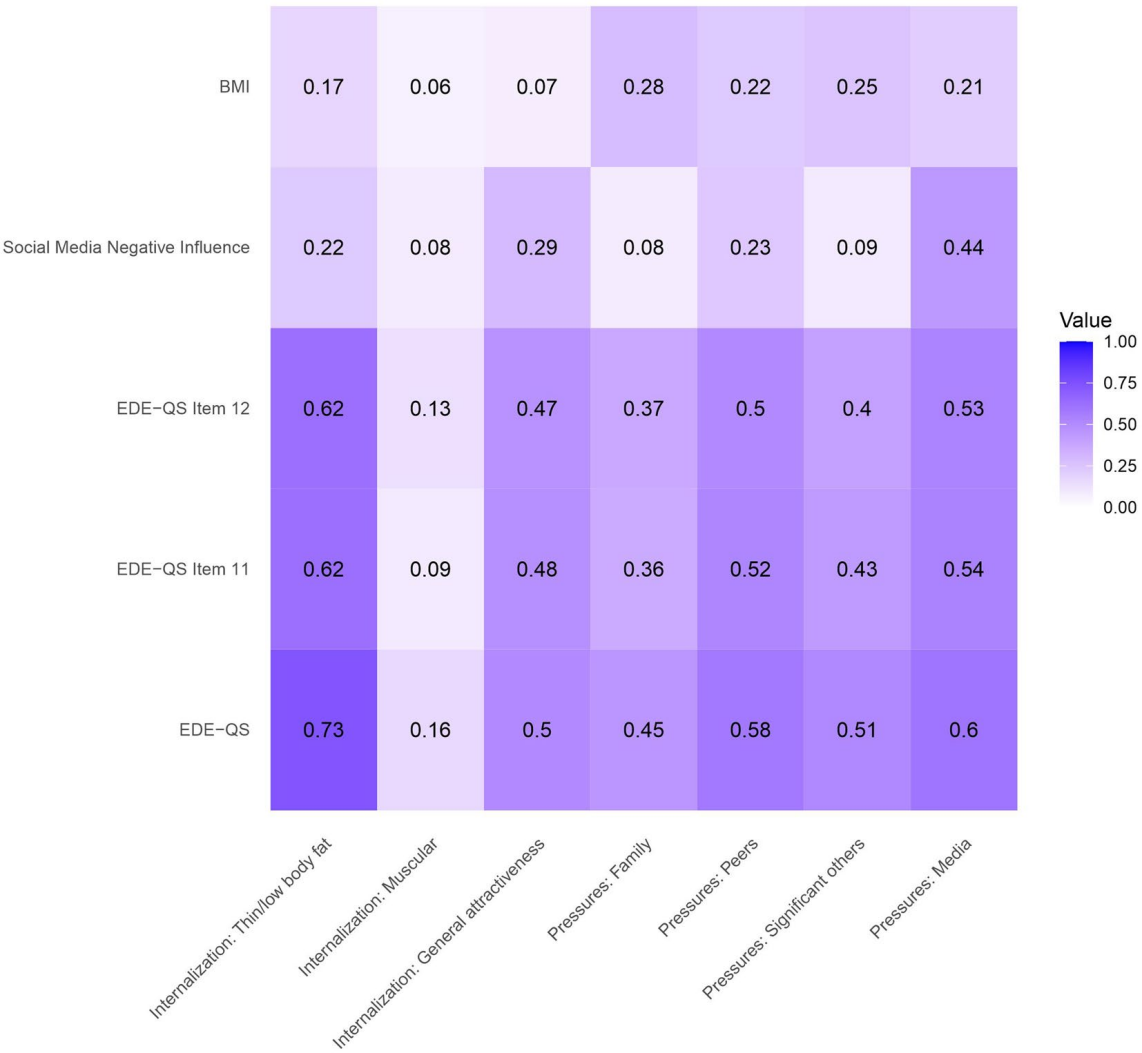
*Correlations (Spearman’s rank or Point-Biserial Correlations) Between SATAQ-4R Female and Convergent Measures. Note.* BMI = Body mass index (kg/m^2^); EDE-QS = Eating disorder examination questionnaire-short; SATAQ-4R = Sociocultural Attitudes Towards Appearance Questionnaire-4-revised. Social Media Negative Influence correlations are calculated using Point-Biserial Correlations, all other correlations are Spearman’s rank. Darker color indicates stronger correlations (closer to 1)

**Fig. 4:**
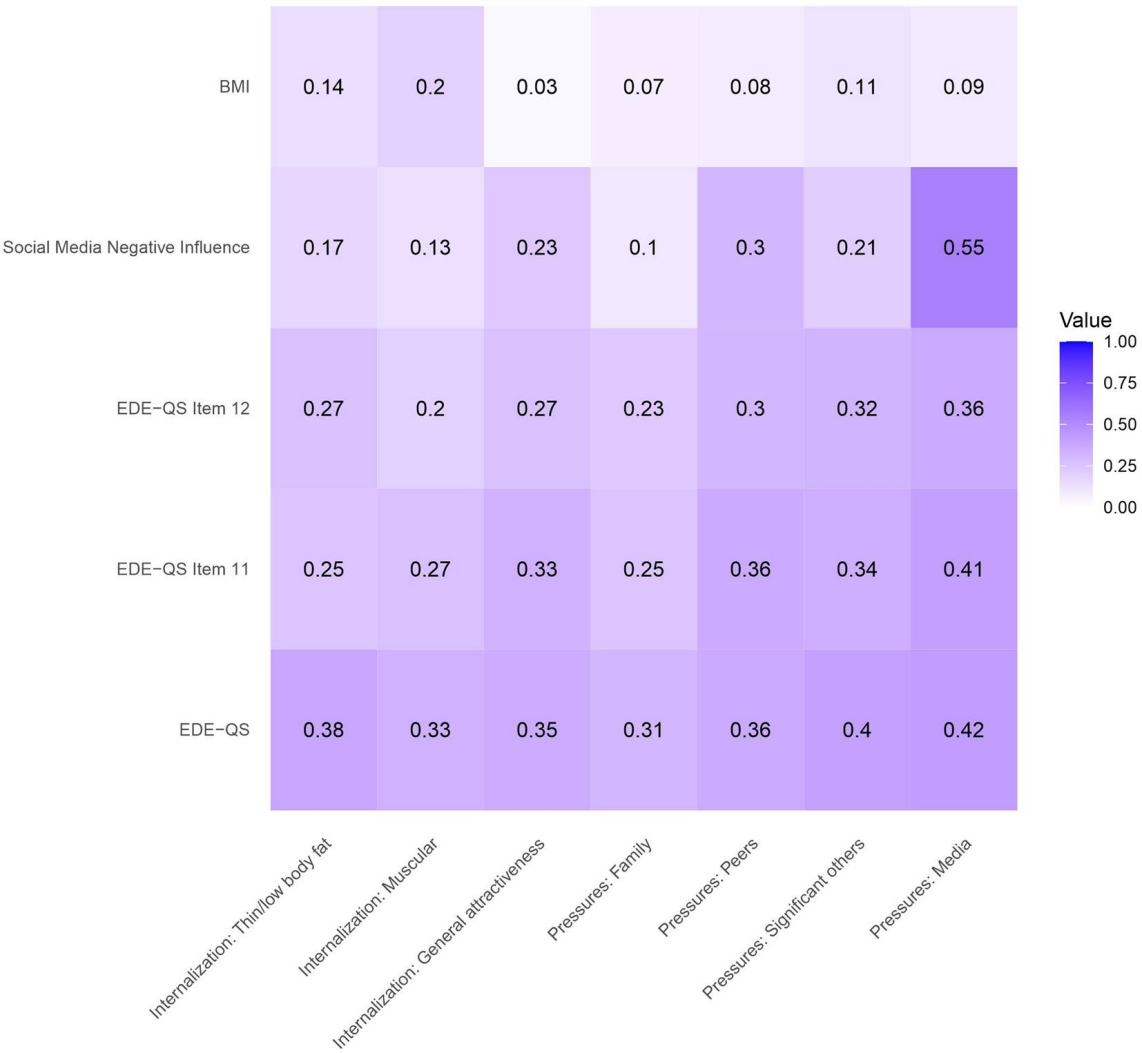
*Correlations (Spearman’s rank or Point-Biserial Correlations) Between SATAQ-4R Male and Convergent Measures. Note.* BMI = Body mass index (kg/m^2^); EDE-QS = Eating disorder examination questionnaire-short; SATAQ-4R = Sociocultural Attitudes Towards Appearance Questionnaire-4-revised. Social Media Negative Influence correlations are calculated using Point-Biserial Correlations, all other correlations are Spearman’s rank. Darker color indicates stronger correlations (closer to 1)

See Figs. 3 and 4 for correlations between SATAQ-4R Female/Male and selected convergent validity measures.

#### EDE-QS

EDE-QS total score was significantly positively correlated with all SATAQ-4R scores for both males and females. Correlations were generally stronger for females than males, except for the correlation between EDE-QS and the *Internalization: Muscular* subscale which was stronger for males. All male and female SATAQ-4R subscale scores were positively correlated with the two EDE-Q items specifically targeting the influence of, and dissatisfaction with weight and shape.

#### Influence of social media

Negative influence of social media was significantly correlated with five subscales for females (Internalization: Thin/low body fat, Internalization: General attractiveness, Pressures: Peers, Pressures: Significant others, and Media) and all subscales for males. The correlations were strongest for Pressures: Media in both females and males.

#### BMI

For females, SATAQ-4R subscales Internalization: Thin/low body fat, Pressures: Family, Pressures: Peers, Pressures: Significant others, and Pressures: Media were positively correlated with BMI. For males, Internalization: Thin/low body fat, Internalization: Muscular, and Pressures: Significant others were positively correlated with BMI.

## Discussion

This article aimed to assess the validity and reliability of the SATAQ-4R measure among male and female Norwegian adolescents. The findings supported a 7-factor model for both females and males. Additionally, all subscales of the Norwegian version exhibited good internal consistency and demonstrated good convergent validity. Overall, the results from the SATAQ-4R were unable to effectively distinguish between clinical ED cases and participants without EDs.

The 7-factor structure of the SATAQ-4R observed among girls in the current study is in line with previous studies among Chinese adolescents aged 13–18 years [38], US college women [35], Italian women [36], and US adult women of sexual minorities [67]. The 7-factor structure showed superior fit indices in the current study when directly compared with the 6-factor structure observed in Turkish college women [37] and US adolescent girls aged 10–14 years [35]. More research is needed to investigate whether age differences, cultural aspects, or other factors contribute to diverse findings of factor structure of the SATAQ-4R among females. Among males, our results support the 7-factor structure, which is consistent with findings in US college men [35], Italian men [36], Chinese adolescent boys [38], and US adult men of sexual minorities [67]. Thus, the literature largely supports the original 7-factor structure for males, while there seems to be more variation among females with support for both the 6- and the 7-factor structures. However, a Brazilian study [68] reported a 5-factor structure in an adapted version for children, including boys and girls aged 7 to 11 years. Overall, as suggested by Huang, Liu [38], the factor structure of the SATAQ-4R may vary by age or sociocultural factors, particularly among girls and women.

In line with previous literature [35–38, 67, 68], appropriate psychometric properties of the Norwegian version of the SATAQ-4R were reported when applied in an adolescent sample. In the current study, all SATAQ-4R subscales demonstrated low accuracy in classifying EDs among females. This is in contrast to Rodgers et al. [69] who compared French clinical ED samples with a college sample and found significant differences in internalization scores. Given these findings and the strong associations between ED symptom measures and SATAQ-4R scores, we anticipated identifying a threshold for ED classification. We can hypothesize that the inability to identify such a threshold in our data may be attributed to the overall high levels of body dissatisfaction among the girls in this study. Another potential factor that could have contributed is the small clinical sample size (*N* = 87).

To our knowledge, this study is the first to attempt to classify ED clinical cases using the SATAQ-4R in female adolescents. Future research should aim to expand on this study by using larger samples to investigate significant thresholds for ED classification.

Consistent with previous literature, the current study reports overall higher SATAQ-4R scores in females, except for the *Internalization: Muscular* subscale. This finding is similar to the original SATAQ-4R development and validation study by Schaefer et al. (2017) conducted among college men and women. Interestingly, the differences between males and females are even more pronounced in the current study of adolescents. However, males score higher than females on the internalization of the muscular ideal subscale, suggesting that males to a larger extent internalize the muscular ideal compared to females regardless of age group. Schaefer et al.’s (2017) sample of adolescent girls had somewhat lower mean scores across subscales compared to our sample (mean age 12 versus 17 years). This may indicate that internalization and pressures relating to appearance increase during the adolescent age period. This is further supported by similar mean scores among Chinese adolescents (mean age 16 years) [38] and Italian adults aged 18–65 years, but with the majority (90%) in the ages 18–30 years [36]. Collectively, internalization and pressures related to appearance appear to escalate during the adolescent years before diminishing in adulthood. Females generally tend to experience higher levels of appearance internalization than males. Although there is some evidence to suggest that these patterns may be consistent across cultures, further research is needed to explore this in greater depth.

### Strengths and limitations

Our study benefits from a relatively large, gender-balanced sample of adolescents and offers new insights into the factor structure of the scale among older adolescents. However, several limitations should be acknowledged. Considering that data collection occurred during the COVID-19 pandemic, it is important to account for the increased use of social media during this time, which likely amplified the internalization of and perceived pressure to conform to prevailing beauty standards. This may have, in turn, contributed to the onset or intensification of eating disorder psychopathology in the current sample. Additionally, we did not include additional measures of internalization or pressure to establish construct validity, a measure of body dissatisfaction would have provided further convergent validity, and we did not perform a test-retest assessment. The ROC analyses were underpowered, as only 87 female participants completed the diagnostic interview, necessitating cautious interpretation of the results. Further, no specific quality indicators were embedded in the online survey, which may have had implications for the validity of the responses. While the EDA-5 has shown good psychometric properties in (young) adults [54], it has not been validated in adolescents. The same holds true for the Norwegian version of the EDE-QS.

Also, only 14.2% of participants reported having an immigrant background. A more diverse sample would have enhanced the broader representativeness of the findings, which may not generalize to the general population in Norway. Moreover, representativeness might also have been limited due to the largely urban areas of the recruited schools. Finally, the cross-sectional design is a considerable limitation that should be taken into account when interpreting the results of this study.

## Conclusion

The findings from the current study indicate that the SATAQ-4R is a valid and reliable measure for investigating the internalization of appearance ideals and associated pressures in adolescents. We have identified the most appropriate factor structure for Norwegian adolescents and demonstrated that the subscale scores align well with measures of DE and social media influence. Given the significance of sociocultural factors in the development of adolescent mental health problems, and the increasing exposure to appearance ideals, a validated tool capturing this phenomenon in this age group is essential. Future studies should incorporate the SATAQ-4R in research where assessing internalization and pressures to conform to societal appearance ideals is relevant. Another application of the measure would be to investigate SATAQ-4R scores in individuals of varying body size to gain insight into how societal pressures and internalization are experienced across the weight spectrum. Future research should also aim to validate the SATAQ-4R in clinical samples in Norway, which could further inform treatment interventions. Results could also be used to inform universal and selective prevention interventions aimed at reducing adverse internalization and appearance pressures, as targeted programs based on these findings have the potential to improve body image and overall mental health among adolescents. For instance, the dissonance-based selective ED prevention program the Body Project aims to reduce thin-ideal internalization and body dissatisfaction. Research on specific areas of ideals, internalization, and pressures may inform refinements in the content of this program targeting specific at-risk individuals such as adolescent girls. And finally, in addition to examining convergent and construct validity, future research on the psychometrics of the SATAQ-4R could benefit from investigating incremental validity in areas such as appearance-related teasing and overweight/obesity. This line of inquiry may provide valuable insights into the specific effects of internalizing Western appearance ideals and the societal pressures to conform to these standards, particularly among vulnerable and marginalized adolescent populations.

## Data Availability

Materials and analysis code for this study are available by emailing the corresponding author. The data are not publicly available due to containing information that could compromise the privacy of research participants.

## Abbreviations

CFA: Confirmatory Factor Analysis
DE: Disordered Eating
ED: Eating disorder
EDA-5: Eating Disorder Assessment for DSM-5
EDE-QS: Eating Disorder Examination Questionnaire Short
ROC analysis: Receiver Operating Characteristics
SATAQ-4R: Sociocultural Attitudes Towards Appearance Questionnaire-4-revised
